# Perspectives on long-term medical management of urea cycle disorders: insights from a survey of UK healthcare professionals

**DOI:** 10.1186/s13023-025-03647-x

**Published:** 2025-03-19

**Authors:** Karolina M. Stepien, Melanie McSweeney, Antonio Ochoa-Ferraro, Roshni Vara, Paul Riley, Megan Smith

**Affiliations:** 1 Adult Inherited Metabolic Disease Department, Salford Care Organisation, Northern Care Alliance NHS Foundation Trust, Salford, M6 8HD UK; 2 Department of Paediatric Inherited Metabolic Disease, Great Ormond Street Hospital NHS Foundation Trust, London, WC1N 3JH UK; 3 Adult Inherited Metabolic Disease Service, University Hospitals Birmingham NHS Foundation Trust, Queen Elizabeth Hospital Birmingham, Edgbaston, Birmingham, B15 2GW UK; 4 Department of Paediatric Inherited Metabolic Disease, Evelina London Children’s Hospital, Guy’s and St Thomas’ NHS Foundation Trust, London, SE1 7EH UK; 5 Nexcea, Glasshouse, Alderley Park, Macclesfield, SK10 4ZE UK

**Keywords:** Urea cycle disorders, Evidence-based practice, UCD survey, UCD treatment, Nitrogen scavengers, Hyperammonaemia

## Abstract

**Background:**

Urea cycle disorders (UCDs) are rare inborn errors of metabolism which impact the body’s ability to detoxify ammonia produced during protein metabolism. In the UK, there is a nationally adopted guideline for the emergency management of hyperammonaemia in UCD patients, however there is no guideline for long‑term management, and treatment decisions are left to the discretion of individual healthcare professionals (HCPs).

**Results:**

Twenty-three HCPs, comprising 13 (57%) metabolic consultants, two (9%) specialist nurses, four (17%) pharmacists, and four (17%) dietitians, participated in interviews to document their attitudes and beliefs regarding the long‑term management of UCD patients, including their current practices, treatment goals, and clinical ambitions. The highest priority for 14/23 (61%) of HCPs was to minimise the risk of hyperammonaemia, however the ammonia level that HCPs advised they aimed for varied significantly, with some targeting above the upper limit of normal. Glycerol phenylbutyrate was the highest ranked ammonia scavenger treatment amongst HCPs for safety, tolerability, duration of scavenging action and reducing patient burden, and HCPs suggested that it would be the first-line treatment in an updated guideline. All prescribing HCPs agreed they would prefer their patients receive a licenced product rather than an unlicensed one for reasons including more reliable supply, greater insurance/legitimacy, and the reassurance of regulatory scrutiny and approval. However, analysis of NHS England’s dispensing data between July 2023 and June 2024 indicated annual spend on nitrogen scavengers of £6.7 million with unlicensed specials accounting for £3 million (45%) of the total. Differences between HCPs in the awareness of clinically relevant characteristics of ammonia scavengers, including their sodium and propylene glycol content, were observed.

**Conclusions:**

To standardise the treatment of UCDs within and between metabolic centres in the UK, there is merit in developing a UK-specific treatment guideline.

**Supplementary Information:**

The online version contains supplementary material available at 10.1186/s13023-025-03647-x.

## Background

Urea cycle disorders (UCDs) are rare inherited metabolic disorders (IMDs) affecting the metabolism of nitrogen and endogenous synthesis of arginine. They can result in hyperammonaemia with resultant cytotoxic brain oedema and potentially death [1]. UCDs are caused by deficiencies in one of six enzymes or two amino acid transporters [2] and have an incidence estimated at 1:35,000 [3]. The enzyme deficiencies result in conditions: ornithine transcarbamylase deficiency (OTCD, OMIM #311250), carbamoyl phosphate synthetase I deficiency (CPSD, OMIM #237300), N-acetylglutamate synthase deficiency (NAGS, OMIM #237310), arginosuccinate lyase deficiency (ASL, OMIM #207900), argininosuccinate synthetase deficiency (ASS, OMIM #215700), arginase deficiency (ARG1D, OMIM #207800) [2].

Severity and age of onset depend on residual enzyme or transporter function and are related to the respective gene mutations [4]. All the conditions have an autosomal recessive pattern of inheritance, apart from OTCD, which is an X-linked disorder [5]. In the latter, hemizygous males are usually affected by a very severe neonatal-onset form or, less frequently, with milder disease course with presentation later in their childhood or adulthood, mainly depending on the OTC residual enzymatic activity [5]. Heterozygous females can also be severely affected, even with only one pathological gene variant [6]. Clinical management depends on the severity of their symptoms at diagnosis.

Medical management comprises controlling dietary protein intake to avoid excessive ammonia production or protein catabolism and the administration of nitrogen scavenger drugs to enable excretion of excess nitrogen via the urine. UCDs caused by mitochondrial, rather than cytosolic, enzyme deficiencies may also warrant treatment with arginine or its precursor, citrulline, because patients can exhibit arginine deficiency related to their specific urea cycle defects [7] For UCD patients, liver transplantation is currently the only curative option [8, 9]. The efficacy of other therapeutic options including mRNA therapy [10] and gene therapy [11] is being evaluated in clinical trials.

Medical management can be challenging because of the variation in disease severity, the risk of metabolic instability, treatment, hospital attendance, non-adherence, and limited options for dose titration with nitrogen scavenging drugs [12]. There is a pan-European guideline on UCD management, but it was last reviewed in 2019 [4] and is out-of-date regarding more recent evidence considering comparative effectiveness and tolerability of different nitrogen scavengers. In the United Kingdom (UK), the British Inherited Metabolic Diseases Group (BIMDG) maintains clinical guidelines for the emergency management of children and adults experiencing metabolic decompensation [13]. Acute hyperammonaemia is managed initially with intravenous glucose, L-arginine and sodium benzoate and/or sodium phenylbutyrate in addition to immediate cessation of protein intake before the oral administration of nitrogen scavengers and amino acids such as arginine and citrulline to aid in maximising the excretion of ammonia [8]. The BIMDG also provides a formulary of medicines for use in the management of people with UCDs and IMDs [8] but has so far not developed guidelines for the long-term chronic management of UCD patients where the priority is to maintain metabolic control, avoid chronic complications, and achieve normal growth and development [14].

To understand factors influencing prescribing decisions made by HCPs in the management of UCD patients, we undertook a survey to (1) understand the attitudes and perceptions of HCPs regarding UCD management, (2) analyse how these attitudes and perceptions influence clinical practice, and (3) assess the degree to which patients are being managed in accordance with the latest published evidence. We also analysed NHS dispensing data across England to gain a sense of the use of different nitrogen scavenger formulations by different metabolic centres. The overarching aim was to understand whether an updated guideline on the medical management of UCDs was warranted.

## Methods

### Participants

Potential participants in the research exercise were identified based upon their involvement in the treatment of patients with UCDs at specialist metabolic centres in the UK. Seventy HCPs across the UK were invited to take part via email and comprised inherited metabolic disease specialist consultants, nurses, pharmacists, and dietitians. The research exercise involved a 45-minute interview via Microsoft Teams during which HCPs were asked survey questions (Supplementary Material 1) and their responses documented. Recruitment to the study ended at the point at which data saturation was achieved; this was when no new relevant knowledge was collected from interviews with additional participants in line with the consolidated criteria for reporting qualitative research (COREQ) guidelines [15].

### Survey design

The survey was designed with qualitative and quantitative questions, to gain insights into the attitudes, beliefs, and behaviours of HCPs treating patients with UCDs (Table 1). Semi-structured interviews were carried out to collect responses regarding respondent demographics and experience, current use of nitrogen scavengers, goals of treatment for UCDs, and clinical ambitions for outcomes. The survey included open questions and Likert scale questions. The interviews were performed in accordance with the British Healthcare Business Intelligence Association (BHBIA) Healthcare Market Research Guidelines [16] and the Association of the British Pharmaceutical Industry (ABPI) Code of Practice [17]. The results of the survey were reported in line with the COREQ guidelines [15].

**Table 1:** Themes used in survey and examples of topics

Themes	Example topics
Demographics	• Role • Number of UCD patients • Years in practice
Goals of nitrogen scavenger treatment	• Priority for goals of treatment • Indicators of successful UCD management • Target ammonia levels
Current use of nitrogen scavengers	• Number of patients on each scavenger • What determines treatment • Dual-scavenger treatment
Differences between nitrogen scavengers	• Ranking attributes of scavengers • Titrating scavengers • Use of specials
Evidence-based prescribing	• Guidelines used • Need for new guidelines
Clinician ambition for outcomes	• Satisfaction with scavengers • Indicators of treatment failure • Achieving outcomes

### Survey data collection and analysis

All available data were collated for analysis. One survey was incomplete because the questions were not answered within the allotted interview time; the data that were collected were nevertheless included in the analysis. Participants were not obliged to answer all questions, so some questions were answered by only a proportion of the study population. The raw data has not been made publicly available due to the sensitivity of the responses. Descriptive statistical analysis was performed for survey questions which included categoric and numeric variables.

### Dispensing data

NHS Business Services Authority dispensing data [18] from secondary care medicines data [19] was collated for all NHS Trusts in England between July 2023 and June 2024 for all formulations and strengths of glycerol phenylbutyrate (GPB), sodium phenylbutyrate (NaPBA), and sodium benzoate (NaBz). Data collected included the quantity and indicative cost of product dispensed. From this, indicative cost per gram was calculated for each formulation.

## Results

### Participant demographics

**Fig. 1:**
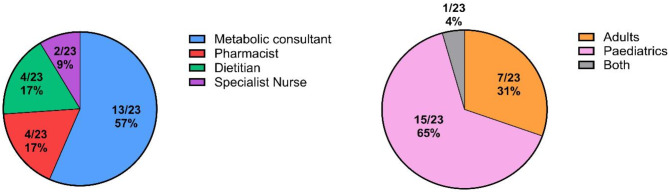
Demographics of the HCPs that took part in the survey. The HCPs represented four different roles involved in the treatment of patients with urea cycle disorders. Participants were involved in the treatment of adult patients, paediatric patients, or both

**Fig. 2:**
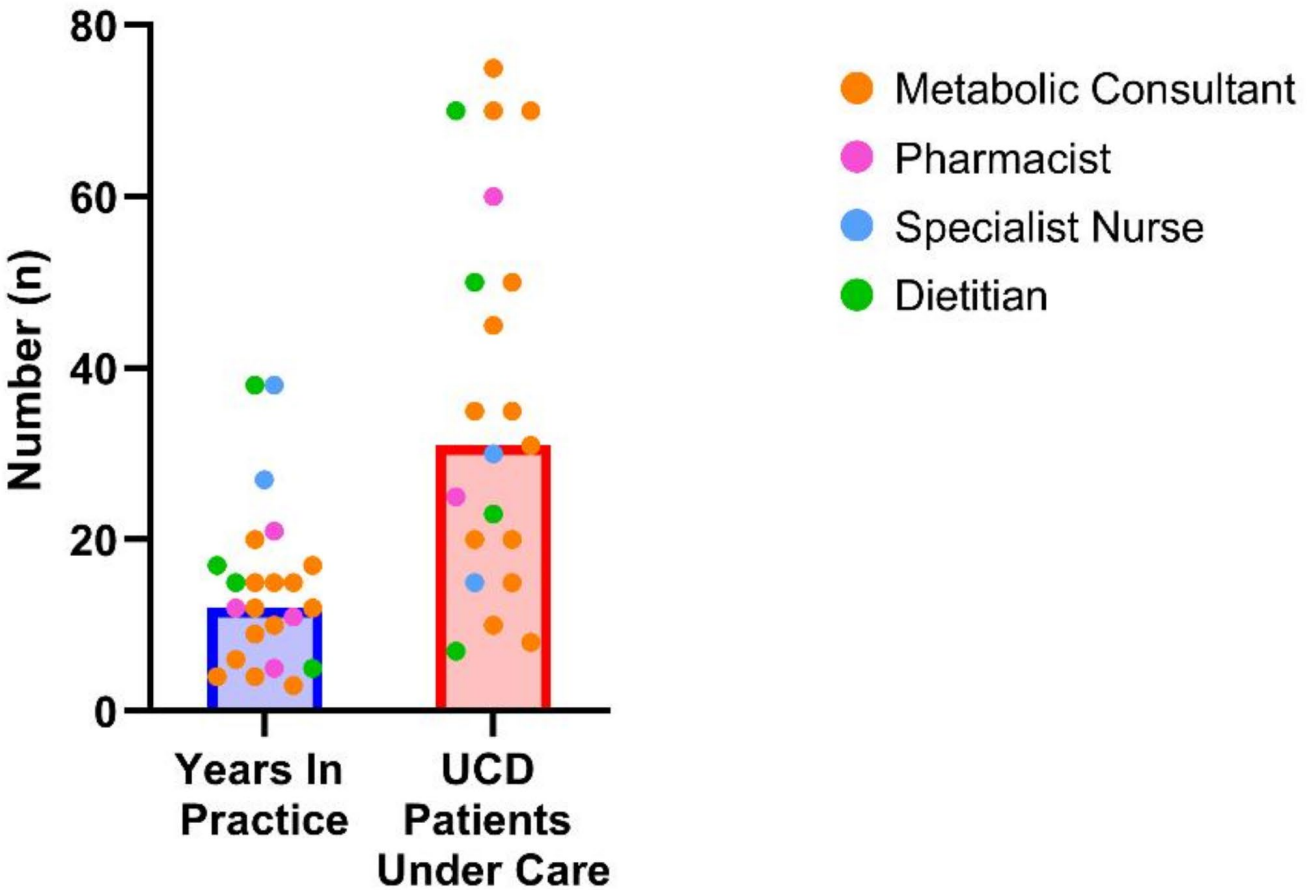
Additional demographics of participants. Bars show median value. The HCPs who participated in the survey had a median of 12 years in practice and were involved in the care of a median of 31 patients with UCDs

A total of 23 HCPs completed the survey (Fig. 1). HCPs varied in their experience of UCDs and in the number of patients under their care (Fig. 2).

### Goals of nitrogen scavenger treatment

When asked about the goals of nitrogen scavenger treatment, the highest priority for most of the HCPs (14/23, 61%) was to minimise the risk of hyperammonaemia. The three indicators of successful UCD management that ranked the highest among participants were (1) an absence of hyperammonaemic episodes/crises (15/23, 65%), (2) optimal patient quality of life (10/23, 43%) and (3) biomarkers within normal limits of the reference range (8/23, 35%). There were no differences in the goals of treatment between HCPs in different roles.

The ammonia level targets that HCPs advised they aimed for varied significantly. For adults, the median target ammonia level was 50 µmol/L, with a range of 20 to 100 µmol/L. For children, the median target ammonia level was 70 µmol/L, with a range of 20 to 100 µmol/L. For neonates, the median target ammonia level was 100 µmol/L, with a range of 20 to 150 µmol/L. Again, there were no differences between HCPs in different roles. Predominantly, HCPs indicated that the target ammonia levels they worked to were based on a combination of the 2019 European guideline, their local guideline, and unpublished but accepted clinical practice. It was also suggested that some patients tolerated higher ammonia levels than others, and target ammonia levels would be higher in those patients accordingly. The UK National Metabolic Biochemistry Network (MetBioNet) guidelines on hyperammonaemia state that for a neonate, typical plasma ammonia levels are < 100 µmol/L, and for infants, children, and adults, < 40 µmol/L [20].

Approximately half of HCPs (12/23, 52%) said that the ammonia targets they aimed for enabled patients to meet their treatment goals ‘to a great extent’, while the other half (11/23, 48%) felt they only enabled patients to meet treatment goals ‘to some extent’. Treatment goals included preserving executive function, minimising the risk of hyperammonaemia, avoiding hospitalisation, and avoiding cognitive damage. More than 50% (13/23) of HCPs advised that they try to keep ammonia levels ‘within the normal range at all times’. However, HCPs also emphasised that there are many factors that can make this difficult, including the disease severity with resulting frequent admissions to hospital, limited response to treatment, limited adherence with treatment and diet, nutritional deficiencies and co-existing illnesses particularly involving vomiting in children, which affects protein intake and catabolism.

### Current chronic use of nitrogen scavengers

The choice of nitrogen scavenger was described as mostly determined by the latest evidence (4/23, 61%) and the changing availability of treatments over time (13/23, 57%). Clinician preference (7/23, 30%) and patient preference (8/23, 35%) were also said to influence prescribing habits. Patient preference was of particular significance for HCPs involved in the treatment of adults, of whom 5/7 (71%) indicated that patient preference determined the choice of nitrogen scavenger.

Most participants (19/23, 82%) believed that the scavenger treatment formulation and/or combination that their patients receive gives them the best chance of achieving the goals of treatment. The four participants who disagreed believed that the available treatments for UCDs are unable to meet patients’ needs, or that liver transplant - and not medical therapy - offered the best outcomes.

The factor that most HCPs considered as preventing patients from achieving the goals of treatment was an inability to tolerate adequate doses of scavenger (10/23, 43%). Alternative reasons included other limitations of scavengers, including their inability to maintain normal levels of ammonia at all times (5/23, 22%). HCPs spontaneously mentioned a lack of treatment adherence (15/23, 65%) and illness (9/23, 39%) as factors preventing patients from achieving treatment goals.

Estimates of the proportion of patients at each treatment centre on more than one nitrogen scavenger ranged from 50 to 100%; dual nitrogen scavenger treatment was reported as being used because patients could not be controlled with monotherapy. Most HCPs (18/23, 78%) considered that being on more than one scavenger was a burden for patients because of the need to take more tablets or greater volumes of liquid, which was regarded as unpleasant because of poor tolerability and unpalatable taste. Despite the acknowledged burden on patients of receiving more than one scavenger, 14/23 (61%) of HCPs said that patients on more than one scavenger could conceivably be changed to monotherapy but as yet have not been.

### Differences between available nitrogen scavengers

**Fig. 3:**
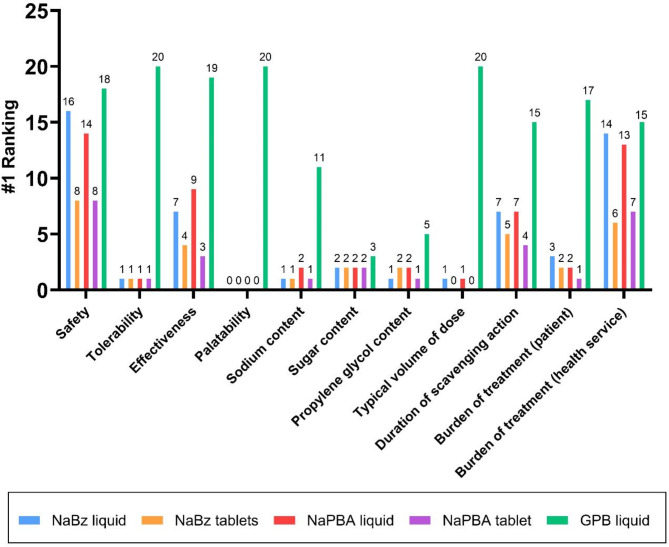
Differences between the available nitrogen scavengers in terms of their patient-relevant attributes. Participants were asked to rank the available scavengers based on what was best for the patient. The number of times each drug ranked as best for patient (ranked as number 1, i.e. best for patient) is shown above the bar. GPB liquid (green) was ranked as best for patient across all attributes. NaBz– sodium benzoate, NaPBA– sodium phenylbutyrate, GPB– glycerol phenylbutyrate

Healthcare professionals were asked to rank nitrogen scavengers based on attributes including tolerability, sodium content, and volume (Fig. 3). Glycerol phenylbutyrate ranked as the highest ranked scavenger treatment for patients across all categories including safety, tolerability, duration of scavenging action, and burden to the patient. Factors determining the dose of scavenger were described as patient bodyweight, severity of disease, and tolerability. It was acknowledged that some scavengers could be titrated to higher doses than others, based on the maximum recommended dose of each scavenger type, and its tolerability. All HCPs said that they would recommend that patients change their nitrogen scavenger treatment if there was evidence that an alternative scavenger may offer better ammonia control, a better treatment experience, and fewer hospitalisations. Certain attributes, including contents of sodium, sugar, and propylene glycol, were not ranked by the majority of HCPs because they acknowledged they did not know how the scavenger formulations compared. This was not the case for any of the pharmacists interviewed, but there were no other noticeable differences between HCP groups.

A significant proportion of HCPs (10/23, 44%) said they used nitrogen scavenger ‘specials’ because there was no licensed equivalent medicine available. Specials are unlicensed medicines manufactured without a marketing authorisation from the Medicines and Healthcare products Regulatory Agency (MHRA). All prescribing HCPs agreed that they would prefer their patients receive a licensed product rather than a special for reasons including more reliable supply, greater insurance/legitimacy, and the reassurance of regulatory scrutiny and approval. Additionally, HCPs advised there are hospital pressures to avoid the use of unlicensed medications.

**Fig. 4:**
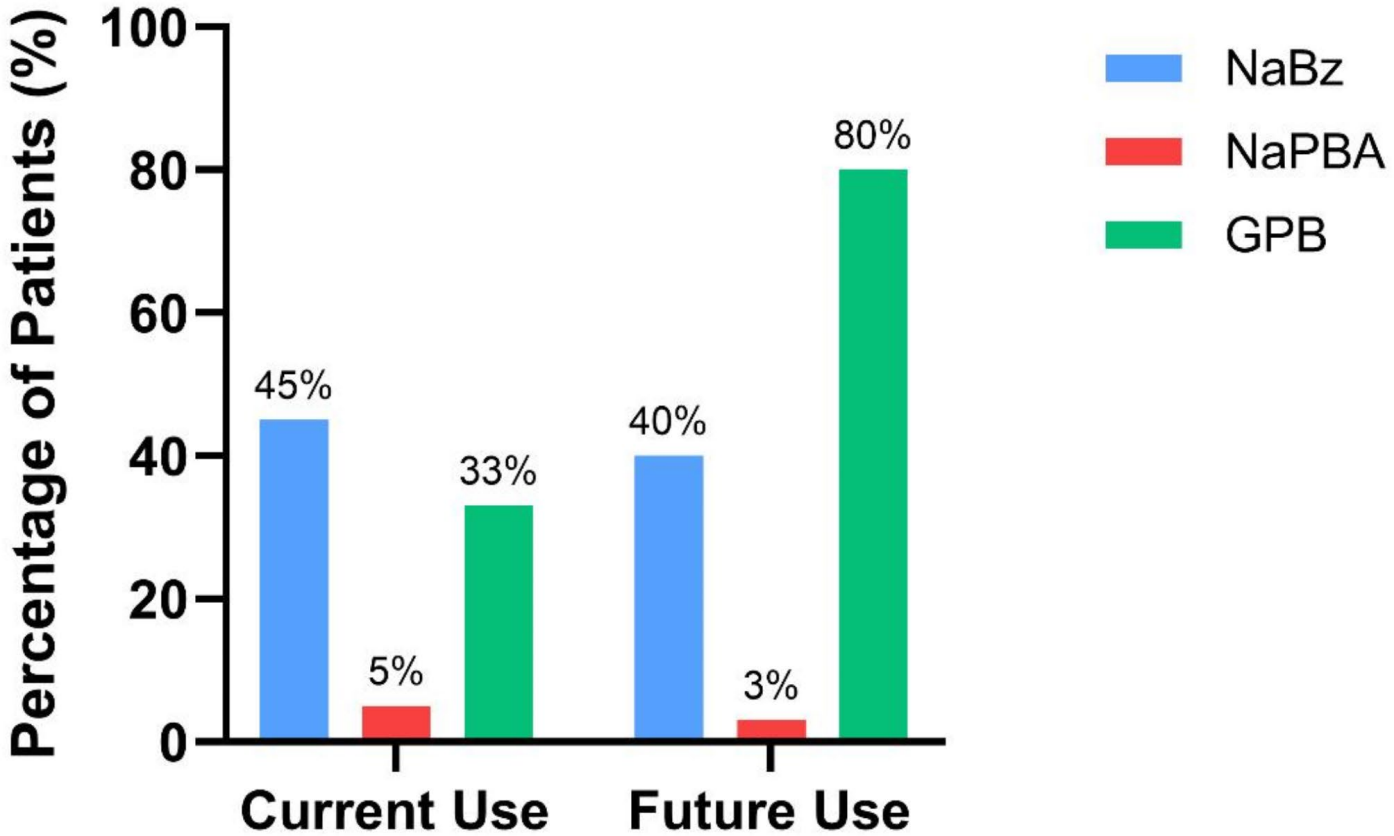
Use of nitrogen scavengers. HCPs indicated that currently, the majority of their patients were prescribed NaBz (blue, 45%). However, if they were to be presented with 100 new UCD patients (future use), the majority of their patients would be on GPB (80%). Bars show median number of patients. NaBz– sodium benzoate, NaPBA– sodium phenylbutyrate, GPB– glycerol phenylbutyrate

When asked to estimate their current use of each of the available nitrogen scavengers, HCPs advised that NaBz was used in a median of 45% (range 0-100%) of their patients, NaPBA in 4% (range 0–70%), and GPB in 37% (range 3-100%). Some patients (median 58%, range 6-100%) would receive dual/combination scavenger treatment. When asked the question, “If you were to be presented with 100 new UCD patients like the ones you currently care for, and you needed to decide on their treatment, what would the numbers look like?”, the 13 metabolic consultants surveyed indicated that GPB would be used in a median of 80% (range 25–100%) of patients, NaBz in 33% (range 4-100%), and NaPBA in 3% (range 0–40%) (Fig. 4); values were higher than 100% as some patients would receive more than one scavenger.

### Evidence-based prescribing

The majority of HCPs (16/23, 70%) believed that there is no need for a UK-specific guideline for the chronic management of UCDs because the BIMDG Formulary and Pan-European guideline are sufficient. Most respondents (20/23, 87%) indicated that they use the Pan-European guideline, 12/23 (52%) use informal local guidelines, and 11/23 (48%) use their own clinical judgement. All respondents (18/18, 100%) indicated that if a guideline was produced today, they believed that GPB would be the first-line treatment, with 10/18 (56%) suggesting that firstline GPB would be supplemented with NaBz where needed.

### Clinical ambition for outcomes

**Fig. 5:**
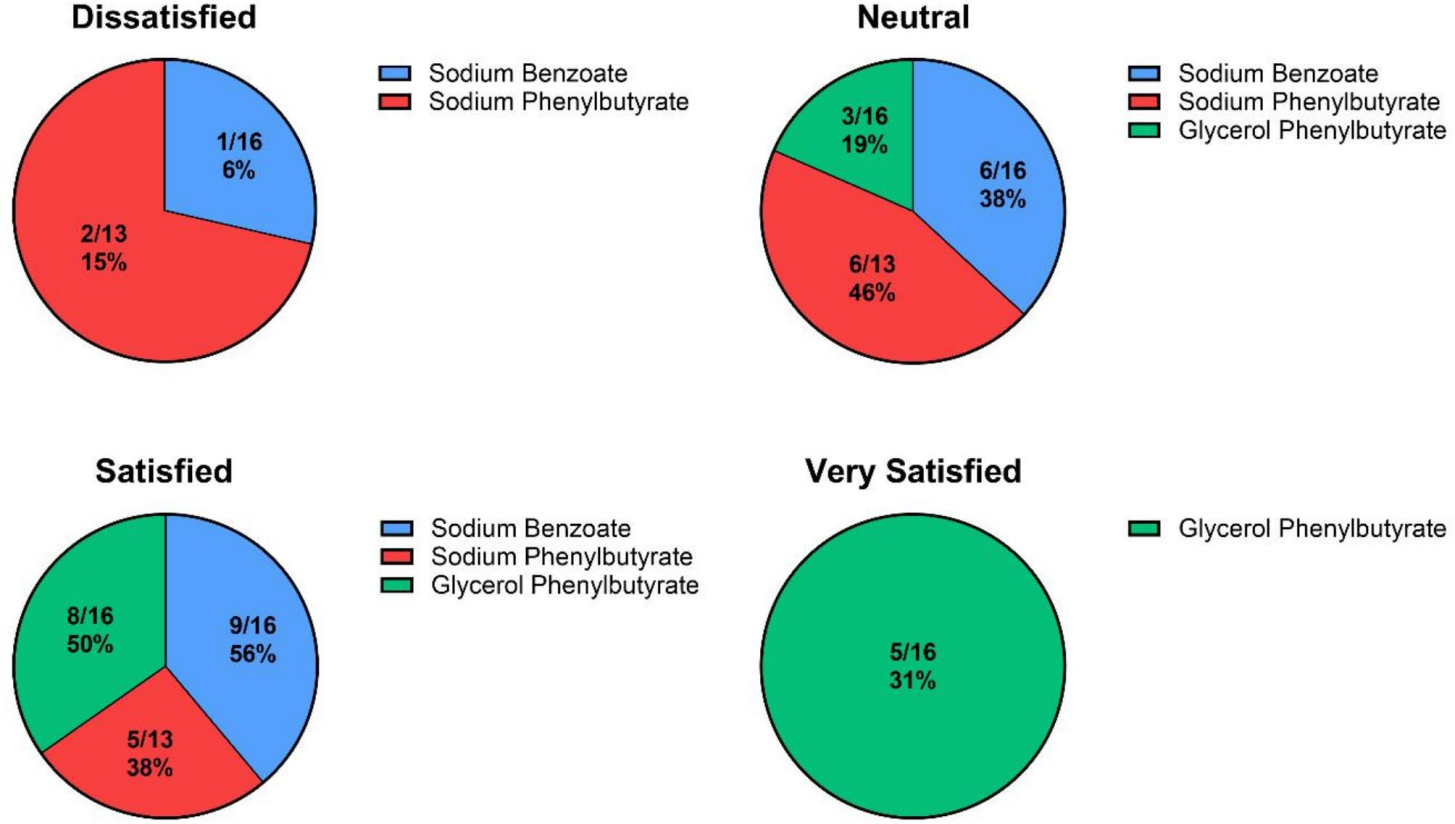
Satisfaction with the level of disease control that can be achieved in UCD patients on different nitrogen scavengers. The number of respondents varied as HCPs were only able to answer based on the scavengers they use in their practice

The majority of participants (14/23, 61%) felt that they were often able to achieve an acceptable level of disease control among their UCD patients, although levels of satisfaction with each nitrogen scavenger varied (Fig. 5).

**Fig. 6:**
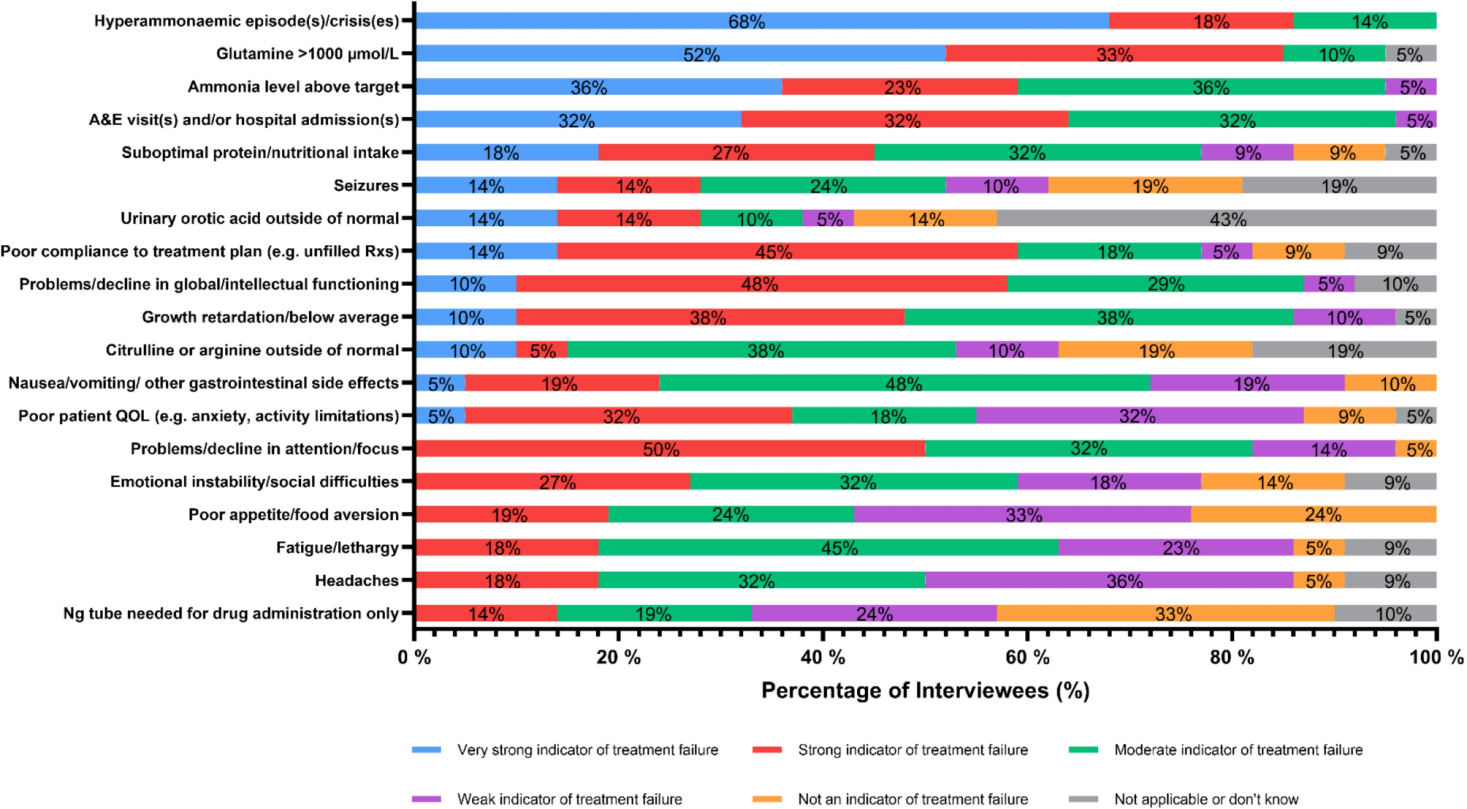
Indication as to what extent the presence of each of the following attributes reflects the potential failure of UCD disease management (*n* = 22). Outcomes are displayed in order, with the attributes that are the strongest indicator of treatment failure at the top of the chart, and attributes that were most often considered to not be an indicator of treatment failure at the bottom

HCPs were asked to consider the extent to which various outcomes indicate failure of UCD management (Fig. 6). Whilst there was reasonable agreement about the strength of some outcomes as indicators of treatment failure, such as hyperammonaemic crises, there were also many outcomes where the opinions of HCPs varied considerably, for example poor quality of life.

**Fig. 7:**
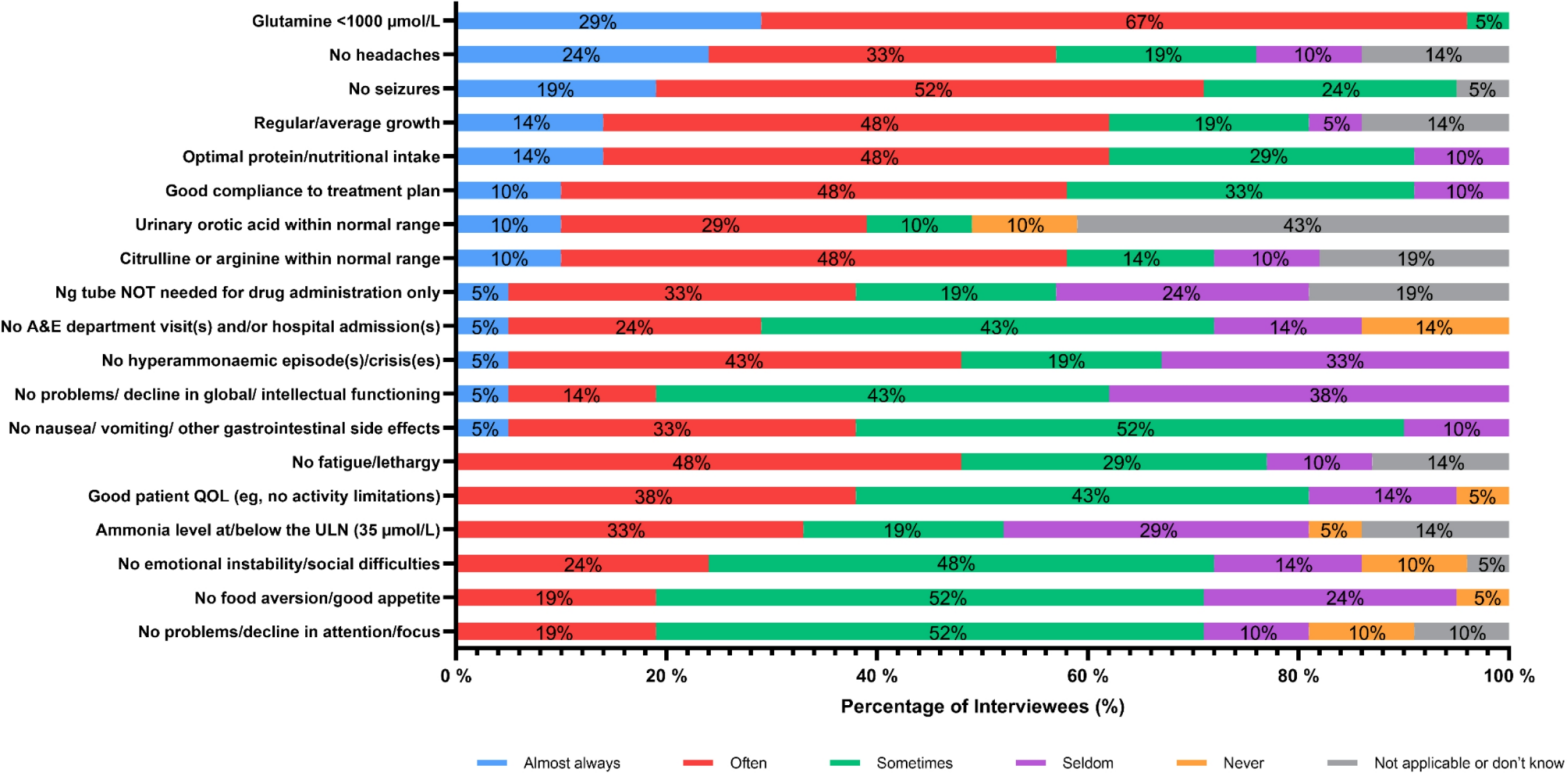
How often certain outcomes are achieved by HCPs in their UCD patients (*n* = 21). Outcomes are displayed in order, with the outcomes almost always achieved at the top of the chart, and outcomes that were indicated to most frequently never be achieved at the bottom

In general, 11/21 (58%) HCPs felt that they could ‘almost always’ or ‘often’ achieve positive outcomes including a glutamine level < 1000 µmol/L, no hyperammonaemic episodes, no seizures, and good adherence to the treatment plan (Fig. 7). Around one-third (7/19, 37%) of positive patient outcomes were regarded by HCPs as being achieved only ‘sometimes’; these attributes included, for example, no gastrointestinal side effects, no problems in functioning, and no food aversions.

### Cost of nitrogen scavengers

The NHS Business Services Authority publishes information about volumes and costs of all medicines dispensed in secondary care to inpatients, outpatients, and via homecare [19]. This dispensing data is publicly available [18]. The data is published monthly, at an NHS Trust level, and can be filtered by product, and further by formulation and strength. The indicative cost per gram of each formulation and strength dispensed at metabolic centres in England was calculated (Table 2).

**Table 2:** All formulations of nitrogen scavengers dispensed in England between July 2023 and June 2024

Formulation	Volume Dispensed (kg)	Indicative Cost (£)	Indicative Cost per Gram (£)
**Licensed Medicines**			
Glycerol phenylbutyrate 1.1 g/mL oral liquid	558	3,268,300	5.85
Sodium phenylbutyrate 500 mg tablets	100	396,078	3.94
Sodium phenylbutyrate 483 mg/g granules	5	19,198	3.94
**Total**	**664**	**3**,**683**,**576**	**5.55**
**Unlicensed Medicines**			
Sodium benzoate 500 mg tablets	173	954,322	5.12
Sodium benzoate 500 mg capsules	33	65,322	2.01
Sodium benzoate 500 mg/5 mL oral solution	294	568,686	1.94
Sodium benzoate 1 g/5 mL oral solution	2	11,140	5.00
Sodium benzoate 1.5 g/5 mL oral solution	33	20,343	0.61
Sodium benzoate powder	10	2,284	0.23
Sodium phenylbutyrate 940 mg/g granules sugar free	84	288,960	3.44
Sodium phenylbutyrate 250 mg/5 mL oral solution	1.13	85,771	75.90
Sodium phenylbutyrate 1 g/5 mL oral suspension	0.02	124	6.19
Sodium phenylbutyrate 1.25 g/5 mL oral suspension	8	74,511	8.88
Sodium phenylbutyrate 1.25 g/5 mL oral solution	50	898,213	18.10
**Total**	**688**	**2**,**969**,**675**	**4.31**

In our survey, four participants (4/23, 17%) indicated that they continued to use unlicensed specials when there are licensed alternatives available, because specials are perceived as cheaper.

## Discussion

Variation in attitudes and perceptions amongst survey participants, coupled with the variation in dispensing data between NHS Trusts, suggests there may be merit in reviewing the evidence base for the medical management of UCDs and updating clinical guidelines. This contention is based on three observations: (1) some information that might influence clinical practice is not commonly known amongst prescribers, (2) there are some contradictions within the survey responses, and (3) the wide variety of nitrogen scavengers in use might benefit from rationalisation.

### Information that might influence clinical practice

All of the HCPs agreed that they would change the oral scavenger treatment of existing patients if there was the likelihood of a better treatment outcome and experience with the alternative scavenger, yet only a proportion of HCPs reported changing the scavenger treatment of existing patients from a sodium-based scavenger to GPB. This was despite HCP sentiment that GPB was a more effective treatment for patients.

Most HCPs were unaware of some of the clinically relevant characteristics of scavengers. Examples included differences in the sodium and propylene glycol content of the various scavengers. A recent publication demonstrated that sodium-based scavengers result in patients being exposed to sodium and propylene glycol above the daily limits recommended for paediatric patients by the NHS and the Royal College of Paediatrics and Child Health (RCPCH) [16, 21, 22]. Chronic exposure to high levels of sodium has been associated with brain dysfunction, confusion, seizures or even death, while propylene glycol can cause central nervous system depression [16]. Propylene glycol can be an excipient in NaBz preparations and is a risk in neonates because it can cause clinical symptoms similar to hyperammonaemia [23].

Differences in survey responses likely reflect differences in clinical practice amongst HCPs. The HCPs expressed a desire to minimise the risk of hyperammonaemia using nitrogen scavengers, whilst aiming for target ammonia levels that were in some instances much higher than the upper limit of normal (ULN). Several HCPs reported that they were comfortable with some patients maintaining ammonia levels above normal, as high as 80 µmol/L or more, because these patients were able to tolerate elevated levels. However, hyperammonaemia can lead to neurological impairment in chronic cases [24] and this can occur over time even in patients with subclinical hyperammonaemia, indicating benefits of tight ammonia control. The lack of agreement between desired outcomes and treatment targets suggests it may be time for targets to be revisited and standardised across the medical community.

Another challenge is related to HCPs describing their use of dual scavenger therapy whilst at the same time acknowledging that using a single scavenger could be feasible. The routine use of dual therapy persists, despite recent evidence from real-world studies indicating that most patients nowadays can be manged on monotherapy with GPB [25, 26]. For some patients with a severe form of the disease more than one ammonia scavenger may be required. However, taking multiple medications, especially those that require more than one dose per day, in addition to the burden of managing different formulations and doses, can have a negative impact on patients’ quality of life, particularly for those with chronic, life-long conditions [27]. In their study, Shchelochkov et al. [21], demonstrated that the biggest barriers to nitrogen scavenger adherence by UCD patients included the number of tablets, frequency of drug administration, and taste of medicines.

### Rationalisation of nitrogen scavengers

Healthcare professionals have a variety of nitrogen scavengers to choose from when making prescribing choices for both the short and long term. All of the HCPs interviewed (19/19, 100%) indicated that they would prefer that patients received a licensed medicine over an unlicensed special. However, the use of unlicensed scavengers in the form of NaBz and liquid NaPBA, as monotherapy and in combination scavenger treatment regimens, was common, accounting for more than half the total amount of nitrogen scavengers dispensed by volume. The MHRA [28], the Royal Pharmaceutical Society (RPS) [22] and the General Medical Council (GMC) [23] provide guidance on the use of specials, advising that they should only be prescribed when the patient has a special clinical need that cannot be met by a licensed medicine of established safety, efficacy, and quality, and that this special clinical need does not include reasons of cost, convenience or operational need [28]. The licensed medicine does not need to be the same molecule as the special; it can be another licensed product in the same therapeutic class [22]. Whilst NaBz and liquid NaPBA may have qualified for use when there were no licensed treatment options, today their use as a monotherapy, despite licensed alternatives, does not align with guidance from the regulator nor professional bodies [22, 23, 28].

Unlicensed medicines are often more costly than licensed medications, and can have longer lead times due to their specialist nature [29]. A 2015 study identified NaBz tablets as amongst the most expensive specials available in the NHS [30]. Data available from the NHS Business Services Authority demonstrated the great variability in the indicative cost of nitrogen scavengers, in particular between unlicensed medicines. Unlicensed formulations of NaPBA vary from an indicative cost of £3.44 per gram to £75.90 per gram. Unfortunately, this variation in price was not discussed during the surveys, and so it is unclear whether clinicians are aware of the true cost of specials. Whilst the present study was primarily concerned with clinical aspects of treatment, it was notable that budgetary concerns were also a feature of decision-making at an individual level, despite those concerns being based on perception rather than evidence.

The average cost per gram of licensed scavengers and unlicensed scavenger specials during the 12-month period analysed was £5.55 and £4.31 respectively. Whilst the average cost of specials was less than that of licensed medicines, there was greater variability in the cost per gram for specials, with costs ranging from £0.23 to £75.90, compared to the much narrower range for licensed medicines of £3.94 to £5.85. Regardless, MHRA and GMC guidance states that cost is not a justifiable reason to prescribe an unlicensed medicine when a licensed medicine is available [23, 28].

In our survey, HCPs ranked GPB as the scavenger best suited for patients. They also suggested it would be first-line treatment in a new guideline. Yet a substantial number of patients still receive NaBz and it featured prominently when HCPs described treatment options for new patients. Some participants mentioned the risks of patients decompensating when changing scavenger treatment, but clinical studies and real-world data from patients switched to GPB suggest this should not be a barrier [25, 26, 31].

### Updating clinical guidelines to reflect the evidence base

HCPs suggested that a guideline specifically addressing longer-term chronic scavenger use is not necessary. Reasons given were that clinical practice already reflects the evidence and that approaches to treatment are uniform across the UK. However, the responses to our survey revealed variation in clinical practice across the UK, and at a local level.

There was a predominant perspective shared by HCPs that, were a guideline to be written regarding the chronic management of UCDs, GPB as scavenger monotherapy would be the first-line treatment. This would be for metabolically stable patients who would likely have been previously treated with intravenous scavengers to gain metabolic control. If there was inadequate metabolic control with GPB monotherapy, NaBz would be added.

### Limitations of the survey

The significant variability in the different UCD types and associated phenotypes made it difficult for respondents to answer some of the questions in a manner that represented ‘the average patient’. Another limitation of the survey was the relatively small sample size - although in accordance with COREQ guidelines [15], recruitment ended after the survey responses were saturated, meaning that there was no new information being collected.

## Conclusions

There would likely be merit in systematically evaluating the effectiveness and tolerability of nitrogen scavengers in the UK, and using the results to devise a UK-specific guideline to help standardise treatment nationally.

## Electronic supplementary material

Below is the link to the electronic supplementary material.

Supplementary Material 1

## Data Availability

The data that support the findings of this study are not openly available due to reasons of sensitivity but are available from the corresponding author upon reasonable request.

## Abbreviations

ABPI: Association of the British Pharmaceutical Industry
ARG1D: Arginase deficiency
ASL: Argininosuccinate lysase deficiency
ASS: Argininosuccinate synthetase deficiency
BHBIA: British Healthcare Business Intelligence Association
BIMDG: British Inherited Metabolic Diseases Group
COREQ: Consolidated criteria for reporting qualitative research
CPSD: Carbamoyl phosphate synthetase I deficiency
GMC: General Medical Council
GPB: Glycerol phenylbutyrate
HCPs: Healthcare professionals
IMDs: Inherited metabolic disorders
MetBioNet: UK National Metabolic Biochemistry Network
MHRA: Medicines and Healthcare products Regulatory Agency
NaBz: Sodium benzoate
NAGS: N-acetylglutamate synthase deficiency
NaPBA: Sodium phenylbutyrate
OTCD: Ornithine transcarbamylase deficiency
RCPCH: Royal College of Paediatrics and Child Health
RPS: Royal Pharmaceutical Society
UCDs: Urea cycle disorders
ULN: Upper limit of normal
UK: United Kingdom
