# Supplementary material for: Perspectives on long-term medical management of urea cycle disorders: insights from a survey of UK healthcare professionals
Source: Orphanet J Rare Dis. 2025 Mar 19;20:135. doi: 10.1186/s13023-025-03647-x (PMC11921535; doi:10.1186/s13023-025-03647-x)
Supplement: Supplementary file 1 — Supplementary Material 1 [file 13023_2025_3647_MOESM1_ESM.docx]

**UCD Management Survey**

**Personal Details**

- Name:
- Role:
- Hospital:

**Demographic Questions**

- How many patients with urea cycle disorders (UCDs) are you involved with the care of?
  - Adults, approximate number ___
  - Children, approximate number ___
- What proportion of these patients attend appointments as scheduled?
  - Adults, approximate number ___
  - Children, approximate number ___
- What is your specialty/role with respect to the care of patients with UCDs? (select one)
  - Metabolic consultant
  - Dietitian
  - Specialist Nurse
  - Pharmacist
  - Other; please specify: _____________________________________
- Years in practice: ________
- Percentage of professional time spent on UCDs: ________

**Goals of ammonia scavenger treatment**

- What are the goals of ammonia scavenger treatment, in order of priority? (You can rank choices as ‘equals’)
  - Minimise the risk of hyperammonaemia
  - Preserve executive function
  - Avoid cognitive damage
  - Avoid hospitalization
  - Other(s) ___
- From the list below, please select the top 3 indicators of successful UCD management, with #1 being the strongest indicator of success.

|  | Rank |
| --- | --- |
| Absence of hyperammonemic episodes/crises |  |
| Absence of A&E visits or hospital admissions |  |
| Biomarkers within normal limits of reference range (eg, ammonia) |  |
| Absence of symptoms of hyperammonemia (eg, nausea, fatigue, headaches) |  |
| Adequate protein/nutritional intake |  |
| Regular growth/development |  |
| Consistent adherence to UCD treatment plan (ie, diet, supplements, medications) |  |
| Normal neurocognitive functioning (eg, attention, executive function) |  |
| Optimal patient quality of life (QOL; eg, minimal limitations on everyday activities; living a “normal” life) |  |
| Adequate performance at school or work |  |
| Other; please specify: _________________________ [70 characters max] |  |

- What target ammonia levels (µmol/L) do you treat to in adults ___ , children ___ , neonates ___
- Why?
  - Following formal EU guidelines
  - Following formal local guidelines
  - Following unpublished but accepted clinical practice
  - Other ___
- Additional information? ___
- To what degree do the targets enable patients to achieve the goals of ammonia scavenger treatment described earlier?
  - To some extent
  - To a great extent
- Ideally what ammonia level should we aim for?
  - Keep patients within normal ammonia range at all times
  - Keep patients within target ammonia range described in guidelines at all times?
  - Other?
- What are the difficulties in keeping patients in the normal ammonia range at all times i.e. below the upper limit of normal, which according to some laboratory reference ranges is 35 µmol/L? ___

**Current use of ammonia scavengers**

- Approximately how many of the patients you said are involved with are on each scavenger? (If patients receive more than one, they should be counted separately for each.)
  - NaBz liquid (unlicensed special) ___
  - NaBz tablets (unlicensed special) ___
  - NaPBA liquid (unlicensed special) ___
  - NaPBA tablets (Ammonaps®) ___
  - Carbaglu® tablets (carglumic acid) ___
  - GPB liquid (Ravicti®) ___
- What has determined the treatment mix?
  - Reflects latest evidence-based evidence regarding the management of patients with UCDs
  - Reflects changing availability of treatments over time
  - Reflects standard local practice
  - Reflects patient preference
  - Reflects clinician’s preference
  - Reflects clinician’s habit
  - Other ___
- Does the treatment mix offer all patients the best possible chance of achieving the goals of treatment mentioned earlier?
  - Yes
  - No
- Explain ___
- What prevents patients from achieving the goals of treatment mentioned earlier?
  - Unable to tolerate high enough doses of scavenger(s)
  - Unable to titrate scavenger dose frequently enough to match needs
  - Available scavengers cannot maintain normal levels of ammonia at all times
  - Other ___
- What proportion of patients are on more than one ammonia scavenger? ___
  - Why?
- Does being on more than one ammonia scavenger present a burden for patients?
  - Yes
    - Why?
  - No
    - Why?

Could patients on more than one ammonia scavenger conceivably be changed to a single ammonia scavenger?

- - Yes
    - How would that be done, what would they be changed to?
  - No
    - Why?

**Differences between the ammonia scavengers**

- How do the available ammonia scavengers differ in terms of their attributes that are relevant to patients? Rank them in terms of best for patient to worst for patient (you can rank choices as ‘equals’).

|  | NaBz liquid (unlicensed special) | NaBz tablets (unlicensed special) | NaPBA liquid (unlicensed special) | NaPBA tablets (Ammonaps^®^) | GPB liquid (Ravicti^®^) |
| --- | --- | --- | --- | --- | --- |
| Safety (number and nature of adverse drug reactions) |  |  |  |  |  |
| Tolerability (typically experienced side effect profile) |  |  |  |  |  |
| Effectiveness (ammonia levels, hospitalisations) |  |  |  |  |  |
| Palatability (reported by patient) |  |  |  |  |  |
| Sodium content |  |  |  |  |  |
| Sugar content |  |  |  |  |  |
| Propylene glycol content |  |  |  |  |  |
| Typical volume of liquid or tablets per dose |  |  |  |  |  |
| Duration of ammonia scavenging action |  |  |  |  |  |
| Burden of treatment (reported by the patient) |  |  |  |  |  |

- How do the available ammonia scavengers differ in terms of burden on the health service of arranging and managing treatment? (Rank them from low to high; you can rank choices as ‘equals’).
  - Burden on the health service
    - Explain your answer

|  | NaBz liquid (unlicensed special) | NaBz tablets (unlicensed special) | NaPBA liquid (unlicensed special) | NaPBA tablets (Ammonaps^®^) | GPB liquid (Ravicti^®^) |
| --- | --- | --- | --- | --- | --- |
| Burden on the health service |  |  |  |  |  |

- What determines the dose of ammonia scavenger you titrate to? ___
  - Could you use lower doses? Explain. ___
  - Could you use higher doses? Explain. ___
- Can you titrate to higher doses with some scavengers than others?
  - Yes
  - No
  - Explain ___
- Would you recommend patients change their ammonia scavenger treatment if there was evidence that the alternative might offer better ammonia control, an improved treatment experience, and a reduction in hospitalisations?
  - Yes
    - Why?
  - No
    - Why?
- Why are specials used when licensed ammonia scavengers are available?
  - Perceived as cheaper?
  - No licensed alternatives to the compound exist?
  - Needed because licensed ammonia scavengers are not effective in all patients?
  - Other? ___
- All things being equal, would you prefer your patients received a licensed product instead of a special?
  - Yes
  - No
  - Explain
- If you were to be presented with 100 new UCD patients like the ones you currently care for, and you needed to decide on their treatment, what would the numbers look like?
  - NaBz liquid (unlicensed special) ___
  - NaBz tablets (unlicensed special) ___
  - NaPBA liquid (unlicensed special) ___
  - NaPBA tablets (Ammonaps®) ___
  - Carbaglu® tablets (carglumic acid) ___
  - GPB liquid (Ravicti®) ___
- Why would things be the same/different? _____________________
- In a patient diagnosed at birth, who has been stabilised with i.v. ammonia scavengers and who now needs to start maintenance ammonia scavenger treatment, which ammonia scavenger(s) would you use?
  - NaBz liquid (unlicensed special) ___
  - NaBz tablets (unlicensed special) ___
  - NaPBA liquid (unlicensed special) ___
  - NaPBA tablets (Ammonaps®) ___
  - Carbaglu® tablets (carglumic acid) ___
  - GPB liquid (Ravicti®) ___
- Explain your choice.

**Evidence-based prescribing**

- The most recent guidelines for the management of UCDs were European guidelines published in 2019. There are no national guidelines specifically for the UK, there is just the BIMDG formulary. Is a UK guideline needed?
  - Yes
  - No
- Explain your choice.
- Which guidelines do you work to when managing UCD patients
  - European guidelines published in 2019
  - Published hospital guidelines
  - Informal locally agreed practice
  - Own clinical judgement
  - Other ___
- If a UK guideline was published today, what would you expect to see as the first-line ammonia scavenger for patients who have been stabilised with i.v. ammonia scavengers and who now need to start maintenance ammonia scavenger treatment?
  - NaBz liquid (unlicensed special) ___
  - NaBz tablets (unlicensed special) ___
  - NaPBA liquid (unlicensed special) ___
  - NaPBA tablets (Ammonaps®) ___
  - Carbaglu® tablets (carglumic acid) ___
  - GPB liquid (Ravicti®) ___
- Explain your choice.

**Clinical ambition for outcomes**

- In general, how often are you able to achieve an acceptable level of disease control among your UCD patients? (select one)
  - Never
  - Seldom
  - Sometimes
  - Often
  - Almost Always
- In general, how satisfied are you with the level of disease control you are currently able to achieve with your UCD patients using sodium benzoate? (select one)
  - Very dissatisfied
  - Dissatisfied
  - Neutral
  - Satisfied
  - Very satisfied
- In general, how satisfied are you with the level of disease control you are currently able to achieve with your UCD patients using carglumic acid? (select one)
  - Very dissatisfied
  - Dissatisfied
  - Neutral
  - Satisfied
  - Very satisfied
- In general, how satisfied are you with the level of disease control you are currently able to achieve with your UCD patients using sodium phenylbutyrate? (select one)
  - Very dissatisfied
  - Dissatisfied
  - Neutral
  - Satisfied
  - Very satisfied
- In general, how satisfied are you with the level of disease control you are currently able to achieve with your UCD patients using glycerol phenylbutyrate? (select one)
  - Very dissatisfied
  - Dissatisfied
  - Neutral
  - Satisfied
  - Very satisfied
- In general, how burdensome are the symptoms of UCDs on the everyday lives of patients and their families? (select one)
  - Not at all burdensome
  - Slightly burdensome
  - Moderately burdensome
  - Very burdensome
  - Extremely burdensome
- To what extent does the presence of each of the following indicate potential failure of UCD disease management?

|  | Not an indicator of treatment failure | Weak indicator of treatment failure | Moderate indicator of treatment failure | Strong indicator of treatment failure | Very strong indicator of treatment failure | Not applicable or  don’t know |
| --- | --- | --- | --- | --- | --- | --- |
| Hyperammonemic episode(s)/crisis(es) |  |  |  |  |  |  |
| Fatigue/lethargy |  |  |  |  |  |  |
| Suboptimal protein/nutritional intake |  |  |  |  |  |  |
| Emotional instability/social difficulties |  |  |  |  |  |  |
| A&E visit(s) and/or hospital admission(s) |  |  |  |  |  |  |
| Problems/decline in attention/focus |  |  |  |  |  |  |
| Headaches |  |  |  |  |  |  |
| Poor patient QOL (eg, anxiety, activity limitations) |  |  |  |  |  |  |
| Ammonia level above target |  |  |  |  |  |  |
| Poor compliance to treatment plan (eg, unfilled Rxs) |  |  |  |  |  |  |
| Seizures |  |  |  |  |  |  |
| Problems/decline in global/intellectual functioning |  |  |  |  |  |  |
| Glutamine >1000 µmol/L |  |  |  |  |  |  |
| Poor appetite/food aversion |  |  |  |  |  |  |
| Ng tube needed for drug administration only |  |  |  |  |  |  |
| Citrulline or arginine outside of normal |  |  |  |  |  |  |
| Nausea/vomiting/other gastrointestinal side effects |  |  |  |  |  |  |
| Growth retardation/below average |  |  |  |  |  |  |
| Urinary orotic acid outside of normal |  |  |  |  |  |  |

- In general, how often are the following outcomes achieved among your UCD patients?

|  | Never | Seldom | Sometimes | Often | Almost always | Not applicable or  don’t know |
| --- | --- | --- | --- | --- | --- | --- |
| Glutamine <1000 µmol/L |  |  |  |  |  |  |
| No nausea/vomiting/other gastrointestinal side effects |  |  |  |  |  |  |
| Optimal protein/nutritional intake |  |  |  |  |  |  |
| Regular/average growth |  |  |  |  |  |  |
| No problems/decline in global/intellectual functioning |  |  |  |  |  |  |
| No hyperammonemic episode(s)/crisis(es) |  |  |  |  |  |  |
| Ammonia level at/below the ULN (35 umol/L) |  |  |  |  |  |  |
| No headaches |  |  |  |  |  |  |
| Good patient QOL (eg, no activity limitations) |  |  |  |  |  |  |
| No fatigue/lethargy |  |  |  |  |  |  |
| No problems/decline in attention/focus |  |  |  |  |  |  |
| No emotional instability/social difficulties |  |  |  |  |  |  |
| Citrulline or arginine within normal range |  |  |  |  |  |  |
| No A&E department visit(s) and/or hospital admission(s) |  |  |  |  |  |  |
| Urinary orotic acid within normal range |  |  |  |  |  |  |
| No food aversion/good appetite |  |  |  |  |  |  |
| Ng tube NOT needed for drug administration only |  |  |  |  |  |  |
| Good compliance to treatment plan |  |  |  |  |  |  |
| No seizures |  |  |  |  |  |  |
